# Traumatic Tetanus—Recovery

**Published:** 1880-02

**Authors:** Henry Van Buren

**Affiliations:** Chicago


CHICAGO.

---

### ARTICLE VIII.

*Traumatic Tetanus.—Recovery.*

The case which I herewith report, is, I think, a typical case of tetanus traumaticus. If a subdivision were to be made, it might be classed under opisthotonos. The symptoms and history which will at once be given, will show that we had general tetanus, with opisthotonos, at times a special and marked symptom of the disease.

William J., male, aged 23, single, of previous good health. On the 27th of September last, had the ball of the thumb of the left hand wounded by a circular saw in motion. The wound upon its surface did not exceed one-half inch in diameter, but was irregular and torn, penetrating to the phalanx, the bone not being injured. The wound was immediately dressed by a surgeon whose name was unknown to the patient. On the evening of the same day the young man called on me complaining of severe pain in the thumb. I removed the dressing and a compound resembling pitch or tar which had been used. After cleansing the wound adhesive straps were applied and the man returned to his work, suffered little inconvenience, and in two or three days the wound was re-dressed in the same manner.

On the 3d day of October, six days after the injury, the patient returned to my office, and in a muffled voice said, "there is something the matter of my jaws, I cannot open them." His face was somewhat swollen, the swelling extending to the neck under the angle of the jaw. I scarcely suspected trismus but was at first disposed to attribute the trouble to parotitis. On the following day, October 4th, I was sent for and found the patient at his home with positive symptoms of tetanus. He was lying stretched out on his back with the muscles of the trunk and extremities rigid. During the night the tetanic spasms had commenced, and at this time they recurred at intervals of about thirty minutes. Nearly the whole muscular system was involved when the paroxysms were the most severe.

The jaws in the first stage of the disease became fixed, with an opening between the incisors of one-eighth of an inch, which was fortunate. The muscles having the greatest rigidity from first to last were the masticatory, those of the larynx, the sterno-cleido mastoid, and other muscles of the neck, those of the chest and the flexors of the upper and lower extremities. The muscles having the most severe tension in the spasmodic contractions were the sterno-cleido mastoid, those of the anterior thoracic region, and of the back, and particularly the flexors of the lower extremities. The spasms of the serrati and other respiratory muscles were clonic, and respiration not seriously disturbed. The flexion of the spinal column was always well marked, when the contractions took place as in opisthotonos. The muscles of the face were not greatly distorted, and the act of swallowing never desperate though always difficult. Photophobia existed throughout; bright light would increase the frequency and severity of the spasms. Sudden and startling sounds would cause the same disturbance. From the second to the seventh day the paroxysms were very frequent, at times without complete intermission. At the end of this time they gradually became less frequent. The pulse never exceeded 110 per minute and generally not above 100. The external appearance of the body was normal in color except the face which was quite red. There was little or no increase of temperature. Bowels constipated and urine scanty.

At the end of the first week the mind was wandering and the the conceptions confused, but entire unconsciousness never. For ten days the patient passed sleepless nights and days, almost no sleep, not exceeding half an hour at any one time. In about twenty-eight days from the commencement of the tetanus the spasms entirely disappeared; the trismus also, leaving the muscles stiffened, and their action more or less imperfect.

The treatment was as follows: October 4th, which was the following day after the appearance of tetanus, we prescribed extract calabar bean in (half grain) 0.33 doses, every two hours; given in a solution of alcohol and water. At intervals between these doses we gave (fifteen grains) 1.00 each of chloral hydrate and potassa brom., in glycerine and water, every two hours. These medicines were given for twenty-four hours, and the pupils closely watched, but no perceptible contraction took place. The spasmodic contractions were somewhat lessened, still they were violent. A blister (emp. canthar), was applied to the back of the neck, and counter irritation along the spine.

October 5th, administered by the stomach as before, extract calabar bean (half grain) 0.33 doses every three hours, and sulph. morphia, grain (one-fourth) 0.16; potassa brom., (grains xx) 1.33; and chlo. hydrate, (grains xv) 1.00, every one, two and three hours, as the violence of the disease demanded, and we were able to control the violence of the spasms to a great degree, lessening their force and frequency. The pupils were now contracted. The opium alone would have caused this. At times the opiate was given in doses so large and frequent as to cause contraction of the pupils to a very minute size.

October 6th and 7th, the calabar bean in less frequent doses was continued, and the sulph. morphia and potassa brom., dropping the chloral. During this period, when the swallowing was difficult, or when it was desirable to produce still greater effects with the opiate, gave frequent hypodermic injections of sulphate of morphia; from (one-fourth to one-half grain) .16 to .33 at each injection.

October 8th, Dr. Norman Bridge and Dr. Hanson visited my patient. Dr. Bridge suggested that we try calabar bean without opium, and closely observe the pupils. This was tried for about twenty-four hours. The contraction of the pupils was less than when opium was administered, and that which we did have may have been caused by previous doses of this narcotic. I need not go further in detailing the management of this case. The treatment was substantially the same to the end of the spasmodic contraction. It was pushed vigorously when the case was desperate, and lessened when the acute stage had passed. Flaxseed poultices were constantly applied to the wound, and it was frequently bathed in warm water. The bowels were moved by enema and potassæ acetas given as a diuretic. A liniment containing one part of croton oil to seven parts of castor oil, was applied externally in the after-treatment to remove stiffness of the limbs, and was applied along the spinal column. The diet was beef tea and cream, and these were taken in large quantities through a straw.

The secret in the management of tetanus, if there be any, is to control the spasms and support the patient. The former requires close attention, and opium, I think, is the agent. Calabar bean may have a curative power by its action on the great nervous centers, but thus far its value is not definitely determined; and while it should be tried in cases where we wish to control these most serious disturbances of the nervous system, still in tetanus, I think our chief dependence is in the opium and bromide of potassa and the liberal support of the patient, with a nutritious diet.

HENRY VAN BUREN, M.D.

CHICAGO.

